# Static Testing of a Bridge Using an Interferometric Radar: The Case Study of “Ponte degli Alpini,” Belluno, Italy

**DOI:** 10.1155/2013/504958

**Published:** 2013-10-09

**Authors:** Devis Dei, Daniele Mecatti, Massimiliano Pieraccini

**Affiliations:** Department of Information Engineering, University of Florence, Via Santa Marta 3, 50139 Firenze, Italy

## Abstract

Ground-based radar interferometry is an increasingly popular technique for monitoring civil infrastructures. In this paper, the static testing of a bridge is reported. It was an 8-span bridge, 297 m long, named “Ponte degli Alpini,” crossing the valley of the Ardo River. The radar has been used for testing a lateral span and a central span. The obtained results present elements of novelty not previously reported in the literature. In fact, some displacement measurements of the lateral span have been affected by a horizontal shift that has to be taken into account for a correct interpretation of the measured data. Furthermore, the measurements of the central span have been carried out with the radar positioned transversally with respect to the bridge deck; this unusual arrangement has allowed for obtaining displacement maps less geometrically distorted with respect to other cases reported in the literature.

## 1. Introduction

The static load test of a bridge is part of a process aimed to verify the capability of a structure to support heavy loads. This test is always associated with the analysis of the materials and the design of the structure. The conventional instrument for measuring the deformation of a bridge under static loads is the optical level sensor. It can measure the relative displacement of some points by referring their movement to a reference point off the loaded span, which is supposed steady during the measurement time. This method does not measure directly the deformation of the beams (the bearing structural elements of the span) but the deformation of the slab, supposing it should be strictly joined to the beams. Unfortunately, the accuracy of the optical level sensor decreases rapidly with the distance. Therefore, in case of a long bridge like the structure under investigation, the reference point for measuring the deformation of the central spans cannot be located off the bridge, but it has to be located on another span near the loaded one. In this case, the assumption of the stability of the reference point may not be verified, as the residual effect of the deformation of the loaded span can be transferred to the neighbouring ones.

The interferometric radar is a sensor increasingly used mainly in dynamic testing of infrastructures [1–10]. Nevertheless, it can be used even for static tests as reported in several papers [11–15]. The unique advantages of this technique are (1) measurement of the displacement component along the direction of view; therefore, its accuracy does not depend on distance alike for optical level sensor and (2) capability to provide a displacement map.

In the case study we have reported in this paper, both were important issues, so radar has been selected for integrating the conventional sensors. 

## 2. The Bridge

The bridge under test is named “Ponte degli Alpini,” located in Belluno, Italy. Large reinforcement works were implemented in order to make the bridge compliant with the most recent antiseismic law. The works finished in 2009, the year after a load test was planned before to open the bridge to heavy traffic (over 3.5 tons).

**Figure 1:**
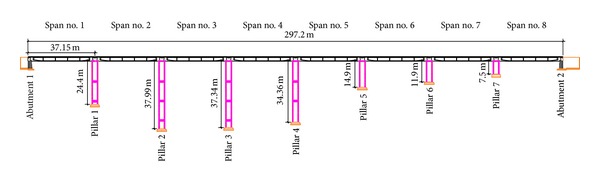
Scheme of “Ponte degli Alpini.”

The “Ponte degli Alpini” is an 8-span bridge, 297 m long. Each span has 4 longitudinal reinforced concrete beams, 37 m long, and a slab. The beams are supported by concrete piers (see Figure 1).

**Figure 2:**
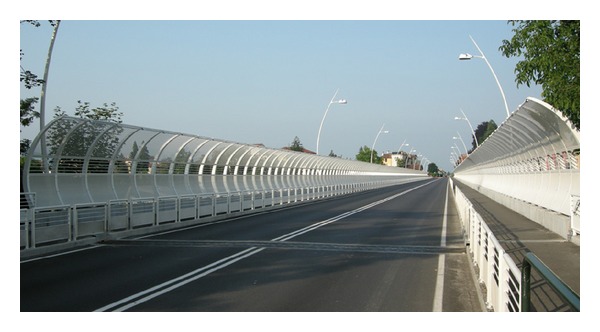
Deck of “Ponte degli Alpini.”

The bridge is 13.40 m large, with a central roadway 9 m large with two sidewalks 2.2 m large as shown in the picture of Figure 2.

## 3. The Lateral Span

**Figure 3:**
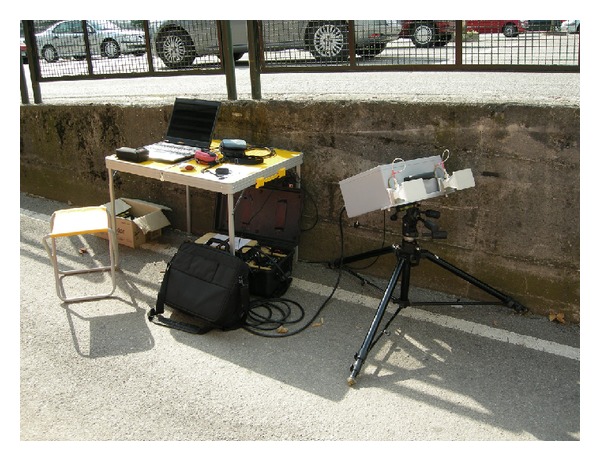
Interferometric radar in only-range configuration.

For testing one of the lateral span (labelled with no. 8 in Figure 1), the radar has been used in only-range modality (see Figure 3). It means that it is able to discriminate the target points only for their different distances from the radar head. 

**Figure 4:**
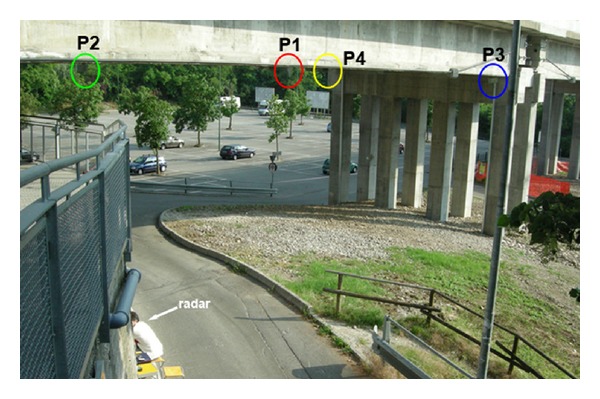
Radar measurement of the lateral span no. 8 in Figure 1.

**Figure 5:**
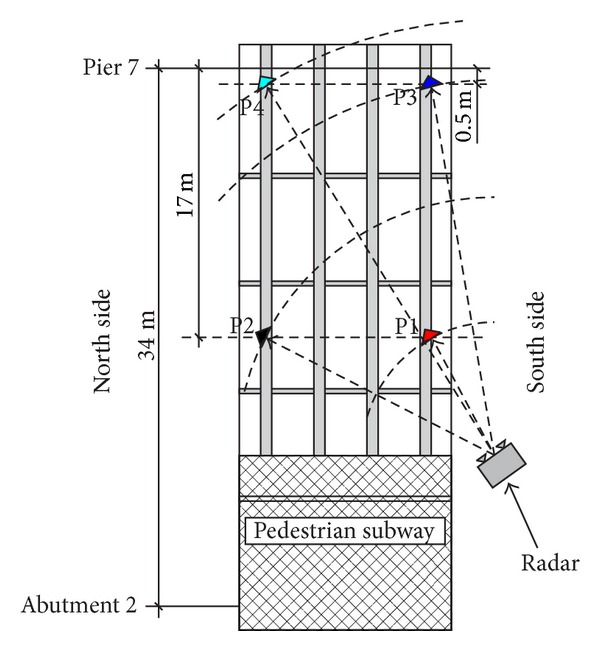
Measurement setup for the spam no. 8.

**Figure 6:**
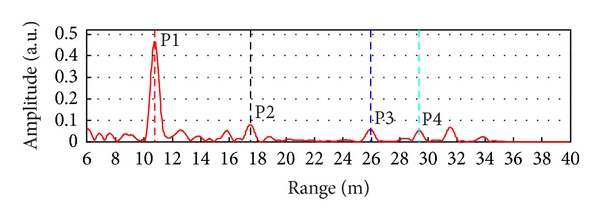
Radar plot relative to the measurement of spam no. 8.

Four dihedral corner reflectors (P1, P2, P3, and P4) have been installed on the lower side of the deck positioned as in Figures 4 and 5. These high reflectivity targets give peaks in the radar plot clearly detectable on the background.  Figure 6 shows the measured radar plot where the 4 corner reflectors are labelled with their numbers.

**Figure 7:**
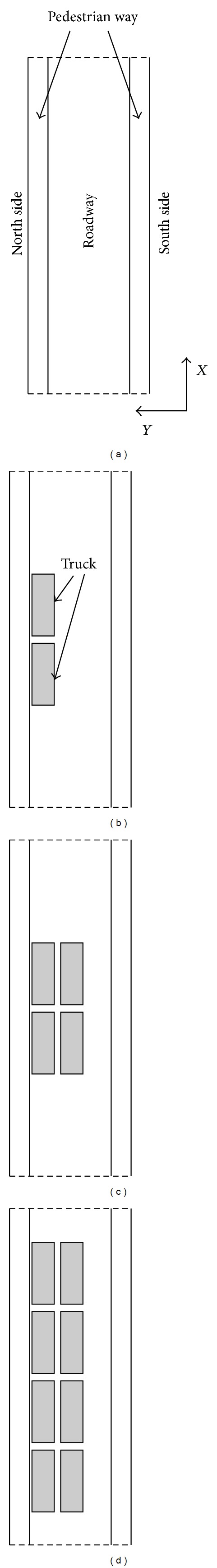
Upward view of the span which illustrates the sequence and the placing of the loads: (a) unloaded; (b) 2 truks; (c) 4 trucks; and (d) 8 trucks.

The load test was carried out by loading the span with 8 trucks. Each truck had 4 axes and was completely filled with sand for a total weight of about 40 tons. The loading procedure is sketched in Figure 7.

**Figure 8:**
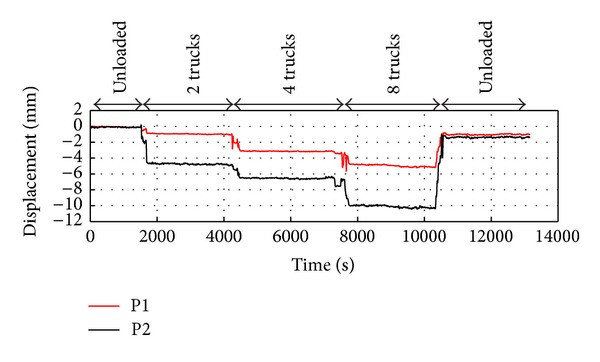
Measured displacements of P1 and P2 during the load test.

In the first loading phase, two trucks were stopped at the centre of the span near the north side, as shown in Figure 7(b). The load was disposed asymmetrically in order to concentrate the effort on one of the external beams. In the second phase, two other trucks were positioned as in Figure 7(c), and in the third phase, the span was loaded with eight trucks (see Figure 7(d)). Finally, the load was removed, and the residual displacement was measured. The radar was set up for acquiring 10 samples per second, so it was able to follow even the dynamic behaviour of the bridge during the different phases of the test. The maximum measured displacement was 10.30 mm ± 0.1 mm (at the P2 point). The relative residual displacement was 1.4 mm (see Figure 8). Both are consistent with the expected values for this kind of bridge. 

**Figure 9:**
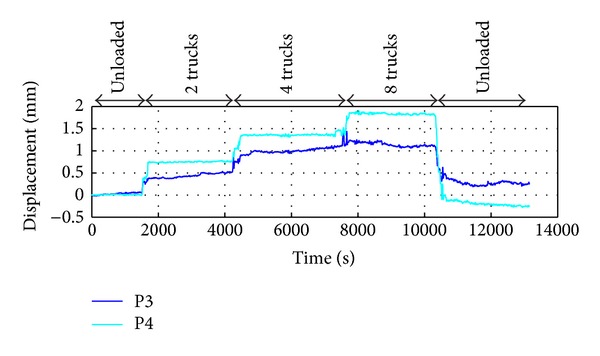
Measured displacements of P3 and P4 during the load test.

**Figure 10:**
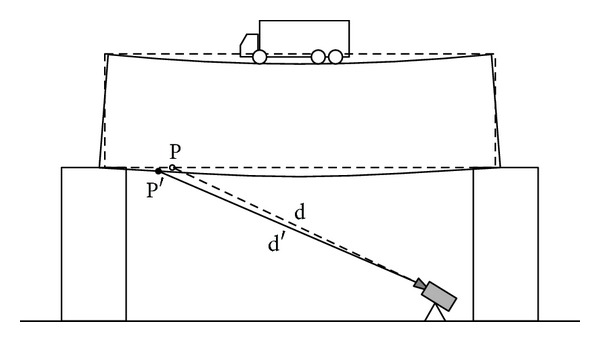
Sketch of the measurement geometry of the points P3 and P4 during the deformation of the deck.

**Figure 11:**
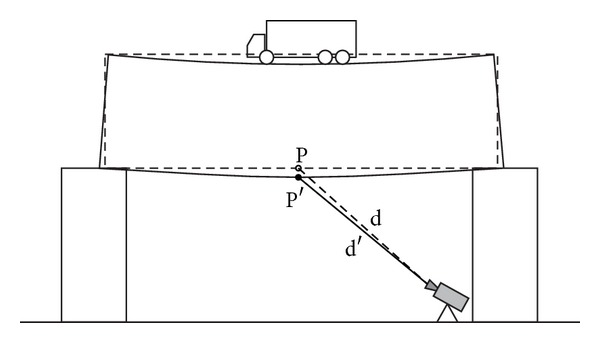
Sketch of the measurement geometry of the points P3 and P4 during the deformation of the deck.

A more interesting behaviour has been observed for points P3 and P4; they gave apparent positive displacements as shown in Figure 9. It is due to the fact that they are not at the centre of the spam but close to the piers. Therefore, during the loading, they move both vertically and horizontally, as shown in the sketch of Figure 10. But the radar see the bridge from a grazing angle, so a horizontal displacement can give a component in view direction larger then the component due to the vertical displacement. This effect is not present for the points P1 and P2 that are close to the spam centre, where there is no horizontal displacement as shown in the sketch in Figure 11.

## 4. The Central Span

**Figure 12:**
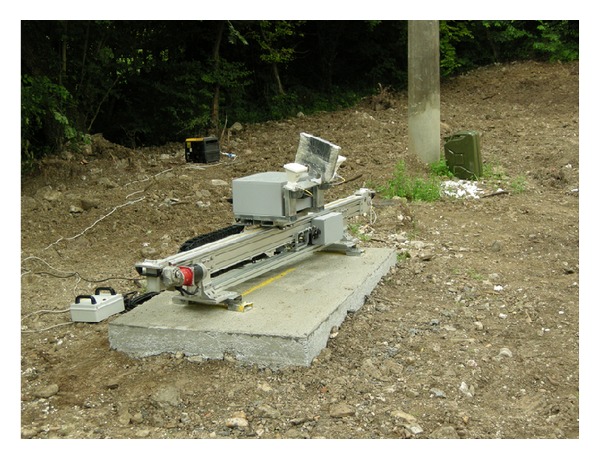
Radar installation on the riverbed.

For testing the central spam (labelled with no. 4 in Figure 1), the radar has been used in cross-range modality. It means that the radar head has been moved during each single measurement on a mechanical linear guide (as shown in Figure 12), in order to acquire a bidimensional image of the deck. In this modality, the radar requires several minutes for a single acquisition.

**Figure 13:**
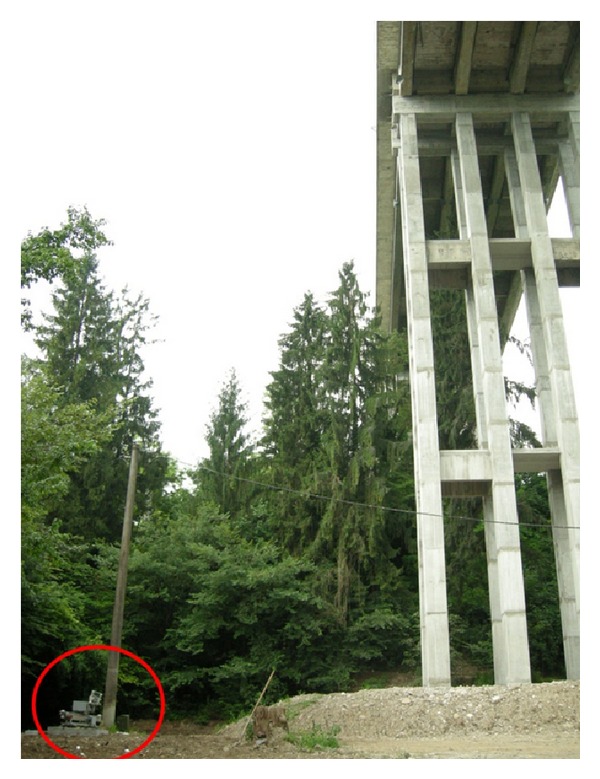
The radar (red circle) placed on a basement under the span.

As the riverbed was accessible, the radar was placed on a concrete basement at ground, 34.5 m below the bridge and 15 m off the span southwards, as shown in Figure 13. The antennas were rotated upward, and they were able to illuminate the whole lower surface of the span. During each measurement, the radar front-end was moved on the mechanical guide with 381 steps of 5 mm to cover the whole guide length of 1.9 m. The radar was set to generate 3057 equally spaced frequency tones sampling a 400 MHz bandwidth, starting from 16.55 GHz. This setting provides 0.37 m range resolution and 0.19 m cross-range resolution at 40 m.

**Figure 14:**
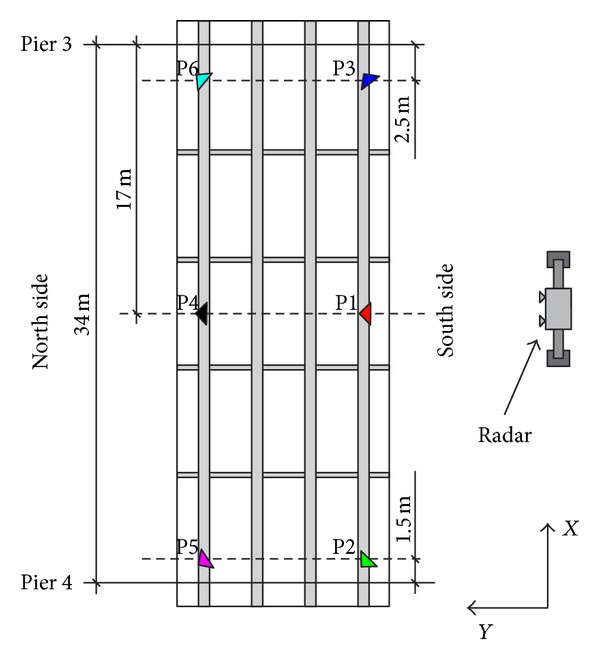
Positions of the corner reflectors (named from P1 to P6).

The static test was carried out with the loading procedure in 4 phases sketched in Figure 7. A first reference measurement was collected when the bridge was unloaded, aftermath, and image has been acquired at each phase. The radar is able to provide a complete displacement image of the lower surface of the bridge, but in order to locate precisely the vertical movement of the points of interest, six trihedral corner reflectors were installed on the external beams as shown in Figure 14.

The interferometric radar in this configuration provides a two-dimensional complex radar cross-section (RCS) images, with amplitude and phase information. The amplitude information is used to identify and select the pixels corresponding to the point of interest. The phase information gives the differential displacement of the identified targets.

**Figure 15:**
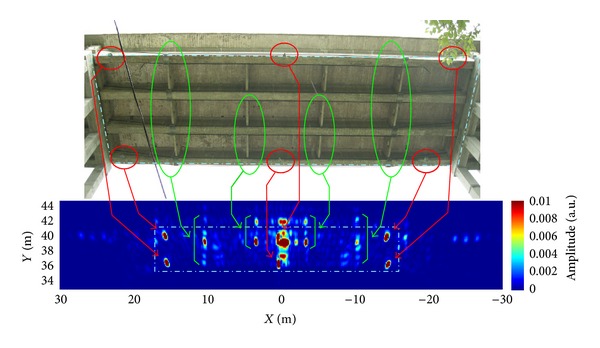
Amplitude radar image with the targets highlighted in the corresponding picture of the span.

The amplitude image of the span is shown in Figure 15. It is an image on the *XY* plane, sampled with increments Δ*x* = 12.5 mm and Δ*y* = 12.5 mm. The *Y* axis is the range, and the *X* axis is the cross-range. The targets highlighted with red circles refer to the corner reflectors and other structural characteristics of the surface.

**Figure 16:**
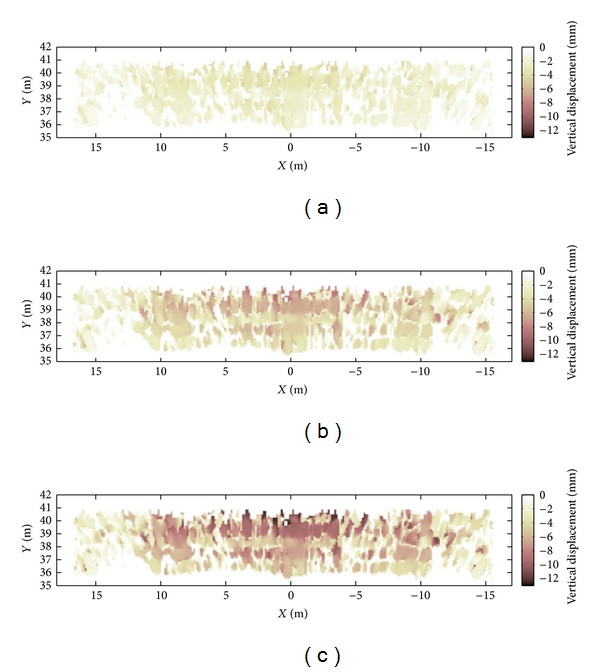
Displacement maps: (a) 2 trucks; (b) 4 trucks; and (c) 8 trucks.

The displacement maps (interferograms) between the reference image, before the loading of the span, and the three following loading phases of 2, 4, and 8 trucks are shown in Figure 16.

The radar image has been masked selecting the pixels with two criteria: (1) a geometrical mask which removes all the pixels scattered out of the surface of the span and (2) an amplitude mask which removes the pixels where the signal is lower than 0.2% of the maximum amplitude of the image. The last one is an empirical method for cutting out the noisy pixels with low backscattered signal. As the radar only detects the displacement component along the line of sight, the vertical displacement has to be calculated by supposing the horizontal component negligible. On the other part, it should be noted that usually the radar is positioned close to a pier with view direction nearly parallel to the bridge span [1, 2, 11, 12]. But in this measurement geometry, a possible horizontal displacement has a large component in the view direction, so it could not be correct to neglect it, as well-evident in the case of the testing of the lateral span reported above. On the contrary, by positioning the radar transversally and at great distance, any possible horizontal displacement in the direction of the length of the bridge has a very small component along the view direction. 

**Figure 17:**
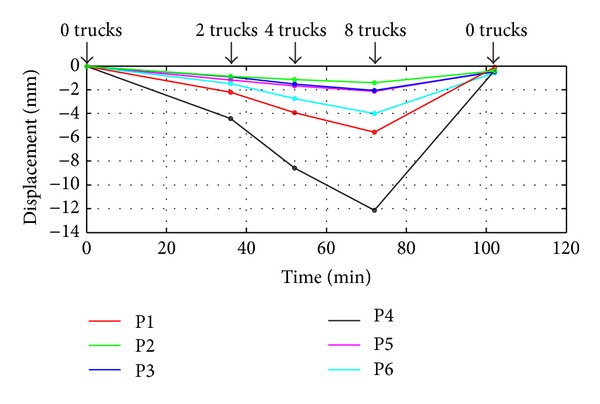
Vertical displacement of the corner reflectors during the loading and the unloading of the bridge: labels from P1 to P6 are referred to in Figure 14.

Finally, Figure 17 shows the calculated vertical displacement of the points corresponding to the corner reflectors installed on the beams, which coordinates have been identified in the amplitude image. The time between a radar acquisition and the next one was as such to allow the structure to settle.

The largest displacement was measured on the beam northward, which was the most loaded, and a residual deformation remains after the final unloading phase as expected. The residual displacement of the six points is reported in Table 1.

## 5. Conclusions

The ground-based radar interferometry has been confirmed as a useful tool for measuring the deformation of a bridge during a static load test. This test is always a key step for assessing the health of a structure and its compliance with the safety guidelines and laws. 

Radar interferometry offers some advantages in comparison with the traditional optical level sensors. The radar can be installed on the ground below the bridge (when it is accessible), and it can detect directly the vertical displacement of the beams. As it overcomes the limit of the optical sensors regarding the accuracy of the measurement when used on long bridge, it can effectively integrate the traditional equipment. 

## Figures and Tables

**Table 1:** 

P1	P2	P3	P4	P5	P6
0.10 ± 0.05	0.40 ± 0.05	0.47 ± 0.05	0.34 ± 0.05	0.44 ± 0.05	0.57 ± 0.05
